# Laser-Driven Rapid Synthesis of Metal-Organic Frameworks and Investigation of UV-NIR Optical Absorption, Luminescence, Photocatalytic Degradation, and Gas and Ion Adsorption Properties

**DOI:** 10.3390/polym16020217

**Published:** 2024-01-12

**Authors:** Saliha Mutlu, Bülend Ortaç, Dogukan Hazar Ozbey, Engin Durgun, Sevil Savaskan Yılmaz, Nergis Arsu

**Affiliations:** 1 Department of Chemistry, Karadeniz Technical University, Trabzon 61080, Turkey; salihamutlu@ktu.edu.tr; 2 National Nanotechnology Research Center (UNAM) and Institute of Materials Science Nanotechnology, Bilkent University, Ankara 06800, Turkey; hazar.ozbey@bilkent.edu.tr (D.H.O.); durgun@unam.bilkent.edu.tr (E.D.); 3 Department of Chemistry, Yildiz Technical University, Davutpasa Campus, Istanbul 34220, Turkey

**Keywords:** metal-organic framework, laser method, photocatalytic activity, adsorption

## Abstract

In this study, we designed a platform based on a laser-driven approach for fast, efficient, and controllable MOF synthesis. The laser irradiation method was performed for the first time to synthesize Zn-based MOFs in record production time (approximately one hour) compared to all known MOF production methods with comparable morphology. In addition to well-known structural properties, we revealed that the obtained ZnMOFs have a novel optical response, including photoluminescence behavior in the visible range with nanosecond relaxation time, which is also supported by first-principles calculations. Additionally, photocatalytic degradation of methylene blue with ZnMOF was achieved, degrading the 10 ppm methylene blue (MB) solution 83% during 1 min of irradiation time. The application of laser technology can inspire the development of a novel and competent platform for a fast MOF fabrication process and extend the possible applications of MOFs to miniaturized optoelectronic and photonic devices.

## 1. Introduction

Metal organic frameworks (MOFs) are composed of metal ions or metal clusters with organic ligands or organometallic complexes. With ultrahigh porosity and large surface areas together with structural tunability and diversity, MOFs can be used in various applications. On the other hand, current synthesis methods should be improved to facilitate the industrial adaption of MOFs and to widen their potential usage. MOFs are a group of porous materials that are increasingly studied and in some fields are well understood. With more than 2000 new articles entering the literature each year, MOFs possess a remarkable level of design flexibility due to their adjustable structures and functions [1]. This enables the incorporation of various inorganic and organic building blocks as well as post-synthetic modifications, resulting in the production of nanoporous materials with diverse functionalities. In recent years, there has been a growing interest in the utilization of MOFs for various liquid-phase applications, including but not limited to, oil refining, aromatic separation, water treatment, solvent recovery, and chemical sensing. Notably, significant advancements have been made in the development of MOFs that exhibit resistance to water and solvents, enabling their successful application in areas such as detection, chiral separation, drug delivery, biomolecule encapsulation, and separation [2]. In addition to industrial applications, such as catalysis [3,4], gas storage [5], separation [6], or sensing, the material class [7,8] of MOFs is also being investigated for diagnostic and therapeutic purposes [9,10,11]. MOF nanoparticles (NPs) for drugs [12,13], nucleic acids [14], peptides, and proteins [15] are currently studied as carriers. Gas separation, in particular, is a crucial industrial process for producing chemicals, fuels, plastics, and polymers. The utilization of porous materials for adsorptive gas separation has promise in meeting the demands of an energy-efficient separation economy [16].

Enhancement of the adsorption and separation capabilities of materials can be achieved by altering metal sites and organic ligands [17,18,19]. At the same time, different chemical components impart significant semiconductor behavior to the framework structure [20,21]. The coordination number of metal ions is very effective in the formation of the crystal structure of the molecule. As an example, Zn(II) d10 is a closed shell metal ion, with three coordination numbers that can take [22], and are suitable for, the construction of various coordination architectures [23]. The energy consumption associated with the transition of the hydrated ion between the three different coordination numbers is around 0.4 kcal mol^−1^ when considered in the gas phase. The ability of Zn(II) to act as a catalytic center is enhanced by its propensity to readily engage in the coordination–decoordination equilibrium, which is contingent upon the prevailing environmental conditions [24]. The zinc ion exhibits the ability to tolerate various structural arrangements, including linear or zigzag chains, ladder configurations, a square, a rhombus, brick wall constructions, and diamond meshes owing to its versatile coordination environment and geometries [25].

In particular, chemical structures with photoactive properties, such as anthracene and its derivatives [26], are very suitable for the process of photocatalytic degradation of organic contaminants. The development of MOFs has garnered significant attention from researchers as a potential solution to the environmental issue of water pollution. This interest stems from the notable characteristics exhibited by MOFs, including their exceptional surface area, porosity, and ability to manipulate their structure and function. The accumulation of pollutants in water poses a significant threat to both the environment and human well-being [27]. Use of the copper-doped zeolitic imidazolate framework-67 (ZIF-67) as a photocatalyst driven by visible light has been observed in the context of degrading methyl orange [21]. According to the findings of Jing et al., ZIF-8 exhibits promising potential as a photocatalyst for the degradation of methylene blue when subjected to UV light irradiation [28].

Currently, several approaches have been employed to fabricate MOFs. Although the solvothermal/hydrothermal synthesis method is frequently used to produce MOFs, various liquid- and solid-phase synthesis techniques are also preferred. These techniques include template synthesis, atomic layer deposition, ionothermal synthesis, spray drying, sol-gel, sonochemical, slow diffusion, conventional heating, electrochemical, and mechanochemical synthesis [29]. In contrast to conventional oven heating techniques, these alternative synthesis methods enabled the large-scale synthesis of MOFs with a significant reduction in crystallization time and, at times, considerable crystal size control [30,31].

Recently, laser-based MOF synthesis has gained prominence. In laser-based rapid synthesis of MOFs, surface growth mechanisms employ lasers, and MOFs themselves serve as the sole substrate. A novel processing technology that primarily employs laser-induced synthesis, the rapid heating method, is distinguished by its capacity for automated manipulation as well as its clean and efficient material synthesis [32]. Laser ablation has the capability to rapidly reduce to an exceedingly high reaction temperature of approximately 4000 K in a matter of nanoseconds (>1010 K s^−1^). This enables the generation and preservation of a vast array of defects, which are valuable for defect engineering purposes [33,34,35,36].

In laser-induced synthesis of MOF derivatives, substances that are more stable and last longer at high temperatures are made possible by the much higher temperature of laser radiation than traditional heating methods. In addition, the densely packed crystal structure of laser-induced MOF derivatives makes them naturally flexible in harsh environments. In recent years, numerous studies on the laser pyrolysis of MOFs have been published, revealing intriguing characteristics of MOF derivatives compared to conventional pyrolysis techniques [37]. Furthermore, the extensive range of MOFs exhibits tremendous potential in terms of uncovering innovative MOF derivatives. As a result, the utilization of laser-processing technology holds immense scientific promise in the realm of MOF derivative preparation and development.

A novel laser-driven synthesis method, distinct from established techniques in MOF synthesis, was illustrated in this research. By introducing organic ligands (H_2_L and bpe) into the aqueous solution of zinc salt, which readily transitions to its ionic state, 975 nm laser beam source heating is achieved. This method is differentiated from laser ablation by the presence of two ligands and the utilization of aqueous salt during synthesis. In contrast to alternative laser fabrication techniques that produce MOF derivatives via laser irradiation of the crystal structure of MOFs, the aim of our research is to create the MOF structure itself in a much shorter time than the solvothermal method using a laser beam, similar to the solvothermal synthesis method. The optical, morphological, structural, photophysical, and photochemical properties of MOFs were examined. Crystalline materials, including MOFs, exhibit notable attributes that are shaped by their chemical properties, defect distribution, and presence. This study presents evidence that defects and the spatial distribution of functions within a multivariable MOF crystal can be precisely evaluated in relation to the overall MOF structure by employing lifetime analysis and fluorescence imaging. The potential to reveal the chemical diversity of laser, optic, morphological, and adsorption techniques used in this study shows that ZnMOF material properties can be understood in a new way, and more successful results can be obtained in MOF synthesis. In addition, ZnMOF synthesized by laser method was investigated in terms of the photocatalytic degradation of MB.

## 2. Materials and Methods

### 2.1. Material Preparations

All reagents were commercially available and used without further purification. 1-(Anthracene-9-methyl)-1H-1,2,3-triazole-4,5-dicarboxylic acid (H_2_L) was synthesized according to the literature [38]. 9-(Chloromethyl)anthracene (98%) was bought from Alfa Aesar, and sodium azide, acetylene dicarboxylic acid, 1,2-bis(4-pyridyl)ethene, Zn(NO_3_)_2_·6H_2_O, DMF (anhydrous), and acetone (extra pure) were bought from Sigma-Aldrich Co., Steinheim, Germany.

### 2.2. Synthesis of Zn-Based MOF [Zn_2_(L)_2_(1,2-Bis(4-pyridyl)ethene)_4_]n

**Scheme 1:**
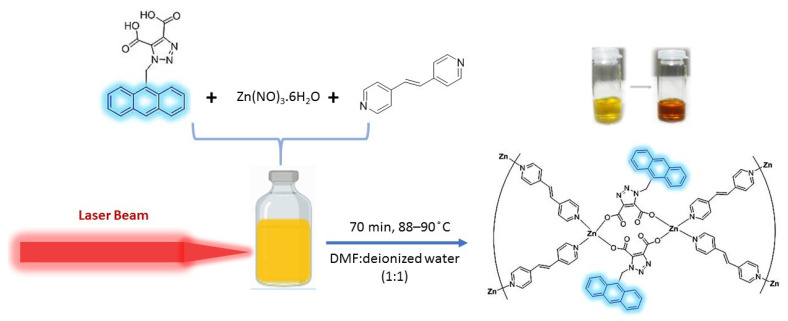
Synthesis mechanism of the ZnMOF structure using a laser technique.

Zn(NO_3_)_2_.6H_2_O (10 mg, 0.033 mmol), H_2_L (11.46 mg, 0.033 mmol), and 1,2-bis(4-pyridyl)ethene (14.04 mg, 0.066 mmol) were mixed in solvents (DMF/H_2_O = 1:1 (*v*/*v*)). The resulting solution was stirred for 5 min. Dark yellow-colored Zn-based MOF crystals were synthesized over 70 min using a laser beam source at 88–90 °C (Scheme 1). IR (KBr): ν = 3054 (br), 1610 (vs), 1424 (m), 832(s), 552 (vs), 408 (m).

### 2.3. Computational Methodology

The computational methodology employed in this study involves density functional theory (DFT) [39,40] calculations implemented within the Vienna Ab initio Simulation Package (VASP) [41,42,43,44]. The potentials for all constituent elements of the MOF molecule were described using the projector augmented wave (PAW) method [44,45]. The exchange-correlation potential was approximated using the Perdew, Burke, and Ernzerhof (PBE) functional within the generalized gradient approximation (GGA) [45]. To correct the underestimated electronic band gaps resulting from the GGA-PBE, the Heyd–Scuseria–Ernzerhof (HSE06) hybrid functional was employed [46,47]. The HSE06 functional incorporated 25% nonlocal Fock exchange, 75% PBE exchange, and 100% PBE correlation energy. A plane-wave basis set with an energy cutoff of 500 eV was used for the calculations. The Brillouin Zone (BZ) was sampled using a Γ-centered 5 × 5 × 5 uniform k-point mesh generated through the Monkhorst–Pack scheme [48]. Periodic boundary conditions were applied along the x, y, and z axes. The atomic positions were optimized using the conjugate gradient method until the forces on each atom converged to less than 0.01 eV/Å. The convergence criteria for the electronic and ionic steps were set to 10^−6^ eV and 10^−5^ eV, respectively. A Gaussian smearing factor of 0.05 eV was utilized. To explore the optical response of the MOF, the frequency-dependent imaginary dielectric function (ε_2_(ω)) was examined using the independent-particle approximation (IPA) [49]. The Kramers–Kronig transformation was applied with a complex shift of 0.01, and the total number of bands was increased to 560.

### 2.4. Photocatalytic Oxidation Studies

The photocatalytic activities of ZnMOF were evaluated using MB. A stock solution of MB (10 ppm) was prepared and used in this study. The solid-to-liquid ratio was kept at 2.5 mg of ZnMOF in 5 mL of MB solution. A Hamamatsu lightning unit, which consists of a 100 W medium-pressure mercury lamp (λ_max_ = 365 nm), was used in the degradation experiments. The ZnMOF and MB solution was placed in a beaker and stirred using a magnetic stirring in the dark for 30 min to achieve adsorption–desorption equilibrium of MB on the catalyst surface. Then, the solutions were irradiated using a medium-pressure Hg lamp for different lengths of time, and the change was examined spectrophotometrically and colorimetrically [16,50,51].

### 2.5. Fabrication and Characterization

A water-cooled diode laser (LIMO GmbH, Dortmund, Germany) was used for the synthesis of ZnMOF. A fiber-coupled CW laser module with 20 W of average output power and 975 nm wavelength was used in the setup. The laser beam source has a power per unit area of 285 W/cm^2^. Two plano-convex lenses were placed in the system in order to collimate the laser beam from fiber output to tightly focused through the ZnMOF platform. UV-Vis-NIR spectra were taken on a Varian Cary 5000 (Markham, ON, Canada). Fluorescence spectra and fluorescence lifetime were recorded on a Horiba Jobin Yvon Fluoromax-P. Infrared (IR) spectra were recorded on a Bruker VERTEX 70 (Billerica, MA, USA) spectrometer α Platinum-ATR spectrometer within the wavenumber range of 400–4000 cm^−1^. ESEM (FEI Quanta 200 F) analysis was performed for ZnMOF to recognize the morphology. An Ametek EDAX energy-dispersive X-ray (EDX) system was used for elemental composition analysis. The crystal phases of the synthesized ZnMOF were characterized using a Multi-Purpose X-ray Diffractometer (Malvern Panalytical X’Pert Pro, Royston, UK) operated at Cu (45 Kv–40 mA) with Cu Kα radiation (λ = 1.54059 Å). An X-ray photoelectron spectrometer (XPS; The Thermo Scientific K-Alpha, Waltham, MA, USA) was utilized to investigate the surface bonding environment of membranes. The XPS spectrum was analyzed using Thermo Scientific Avantage software (v5.9916). N_2_ adsorption–desorption analysis was performed on a QUANTACHROME instrument, Boynton Beach, FL, USA. ZnMOF was degassed at 60 °C for 24 h under vacuum before N_2_ gas adsorption–desorption measurement. A Hamamatsu Lightning cure LC8 (power of lamp: 100 W, intensity of UV light: 241 W/m^2^) was used as the light source for photochemical reactions. The residual concentration of the Pb^2+^ in the solution was determined using Thermo Fisher Scientific XSeries 2.

## 3. Results

### 3.1. Material Synthesis of Laser-Driven ZnMOF and Characterization

ZnMOF structures (classified as ZnMOF2 in reference [38]) were synthesized previously by Biswas et al. using a well-known solvothermal method at 110 °C in 1 day. Additionally, in the literature, a laser ablation technique using metallic zinc and a single organic ligand [52] was found for the preparation of MOF structures. One needs to clearly state that the laser-driven approach that we used is different than the ablation method, the metal source was used in ionic form, and the MOF was synthesized by combining two organic binders via laser. In this study, 1-(Anthracene-9-methyl)-1H-1,2,3-triazole-4,5-dicarboxylic acid (H_2_L) and 1,2-bis(4-pyridyl)ethene (bpe) were added to a [DMF/H_2_O] mixture, and then a laser-driven method with a laser beam with a wavelength of 975 nm was used for the synthesis of ZnMOF, forming a flowery image during 70 min of irradiation, as shown in Scheme 1.

**Figure 1:**
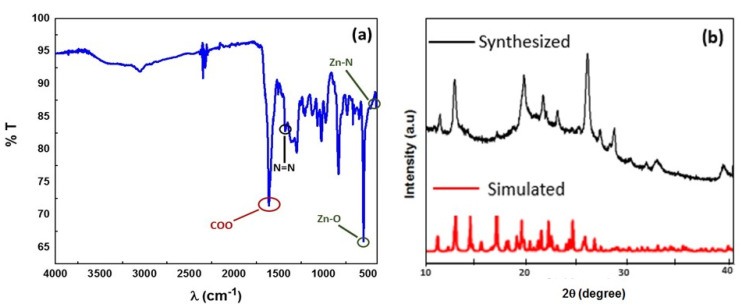
FTIR spectrum (**a**) and XRD result (**b**) of the ZnMOF structure.

Figure 1a shows the FTIR spectrum of ZnMOF. Characterization of ZnMOF involved utilization of the FTIR spectroscopic technique, which revealed a prominent peak at around 408.0 cm^−1^, indicating the presence of Zn-N stretching vibrations. The peak at 552 cm^−1^ indicates the Zn-O bond. The band at 1027 cm^−1^ represents C-O-Zn stretching of the MOF, which shows almost bidentate behavior of the COO group. The bands at 1424 cm^−1^ and 1610 cm^−1^ belong to C-C (in the ligand) and C=O stretching vibrations, respectively. The observed peak at 970 cm^−1^ can be attributed to either the stretching vibration of the C-N bond in the ligand or the stretching vibration of the C-O bond; 1503 cm^−1^ is related to the C=C group of the 1,2-bis(4-pyridyl) ethene ring. The increase in the relative peak density of Zn-N and Zn-O (408 cm^−1^ and 552 cm^−1^) is associated with increased ZnMOF content. Strong Zn-N stretching vibration at 408 cm^−1^ and Zn-O stretching at 552 cm^−1^ show evidence confirming the successful synthesis of ZnMOF.

In addition, binary MOFs of H_2_L (COO ended) with Zn(NO)_3_.6H_2_O and bpe with Zn(NO)_3_.6H_2_O were synthesized. In the supporting information section, the contents of their synthesis are included. FTIR spectra of H_2_L(MOF) and bpe(MOF) structures are given in Figure S1A,B.

As seen in Figure 1b, the notable peaks at 2θ = 12.81° (11−1), 19.58° (200), 21.55° (−10 3), 23.05° (0−30), 25.02° (−203), 25.81° (21−3), and 33.50° (14−2) in the XRD pattern of the crystal ZnMOF show the characteristic diffraction peaks. These results are consistent with the results in the literature [38]. Amorphous formations are due to the H_2_L structure. When the XRD spectra of binary MOFs are examined, H_2_L(MOF) has an amorphous structure (Figure S2A), while the crystalline structure formation is more in bpe(MOF) (Figure S2B). These results show the difference between the ZnMOF structure and binary MOF formations.

**Figure 2:**
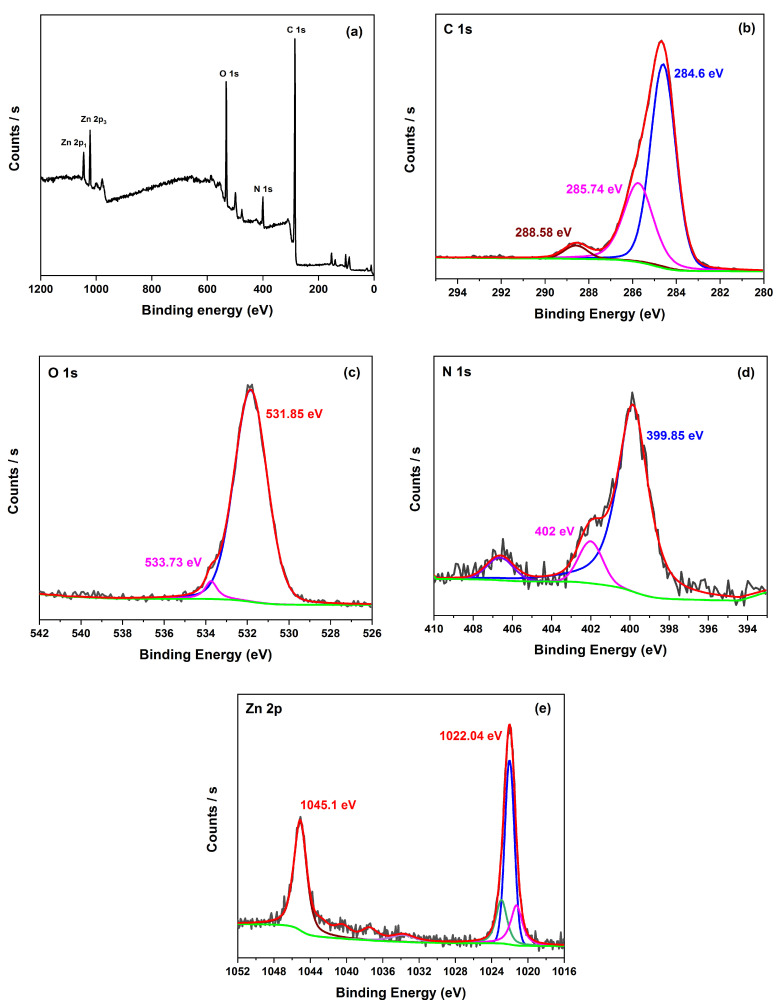
XPS spectra of (**a**) the survey spectrum, (**b**) C 1s, (**c**) O 1s, (**d**) N 1s, and (**e**) Zn 2p of ZnMOF.

XPS analysis was performed to investigate the chemical bonding states in the ZnMOF. Figure 2b shows the C 1s region of the XPS spectra of ZnMOF. The spectra 284.6, 285.7, and 288.58 binding energies refer to C–C/C=C, C–O, and C=O/O–C=O [53,54]. According to Figure 2c, the ZnMOF O 1s spectra can be attributed to two different chemical conditions, C=O and O–C=O, respectively, with two bond energies at 531.85 and 533.73 eV [55,56,57]. Figure 2d depicts the N 1s spectrum of ZnMOF, the binding energy located at 399.85 eV is typical of N–Zn/N–C bonds, and 402 eV could be attributed to N–N bonds [58,59,60]. Additionality, nearly 406 eV shows π excitation of N [61]. Finally, Figure 2e shows that the XPS spectrum of Zn consists of five peaks at 1021.26, 1022.02, 1022.92, 1033.75, and 1045.1 eV, which is attributed to an electron cloud distribution of 2p orbitals. The binding energies of Zn 2p_3/2_ and Zn 2p_1/2_ are located at 1022.64 eV and 1045.09 eV, respectively, with a connection energy difference of 23.06 eV, which corresponds exactly to ZnO. Also, 1021.6 eV can be attributed to Zn–N [57,59,60,62,63,64].

**Figure 3:**
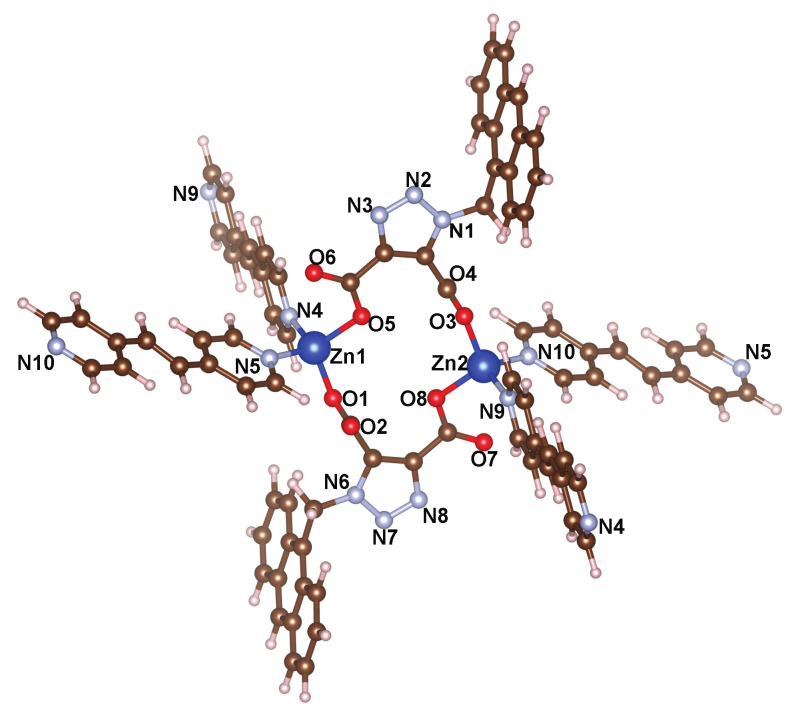
The structure of ZnMOF. The dark blue, light blue, red, brown, and white spheres represent Zn, N, O, C, and H atoms, respectively.

In parallel with the XRD analyses, the ab initio computational calculations show that ZnMOF forms a crystalline structure with a triclinic lattice belonging to the P1 space group (Figure 3). The structure is composed of two Zn atoms, two anthracene triazole dicarboxylate ligands, and two 1,2-bis(4-pyridyl)ethene linkers. The Zn-Zn, Zn-O, and Zn-N distances are calculated as 5.82 Å, 1.98 Å, and 2.07 Å, respectively. Based on Bader charge analysis, it is found that each Zn atom donates 1.21 |e| to the nearest N and O atoms. The obtained framework repeats itself in all dimensions to form the 3D structure (Figure S3). The unit cell dimensions are a = 9.7 Å, b = 11.9 Å, and c = 12.8 Å with 2θ = 96.7°, 2θ = 106.7°, and 2θ = 93.1°.

As a result, ZnMOF, which was produced for the first time in a short time by using metal ions rather than metal from solution with a laser, was compared with other methods in the literature. Table 1 shows a comparison of the duration of the laser synthesis method and the solvothermal method of MOFs.

### 3.2. Photophysical Properties

**Figure 4:**
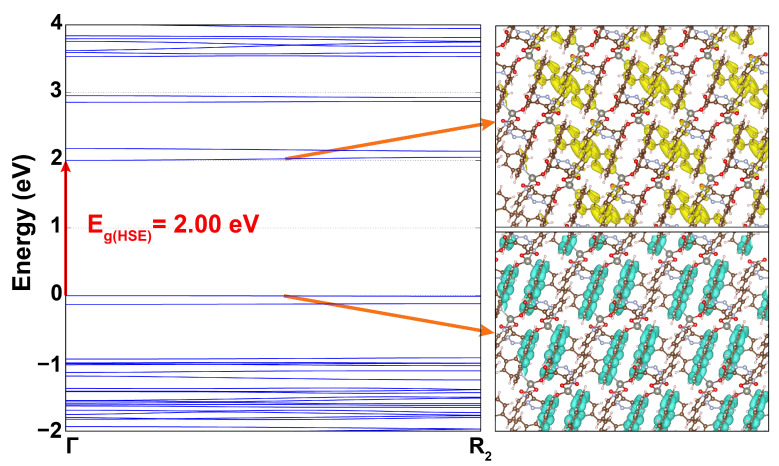
The representative electronic band structure of ZnMOF and band decomposed charge density of valence band maximum (blue) and conduction band minimum (yellow). The electronic band gap is indicated with a red arrow.

Before analyzing the optical response, we calculated the electronic band structure of 3D ZnMOF. The full band structure is shown in Figure S4, and a representative section is illustrated in Figure 4. As can be noticed from the band structure profile, the bands are almost flat along all symmetry directions, indicating a character resembling the molecular level. The band gap calculated at the level of hybrid functional (HSE06) is 2.00 eV. The band decomposed charge density analyses indicate that the valence band maximum (i.e., HOMO) and conduction band minimum (i.e., LUMO) are localized on the anthracene unit and 1,2-bis(4-pyridyl)ethene ligand, respectively.

**Figure 5:**
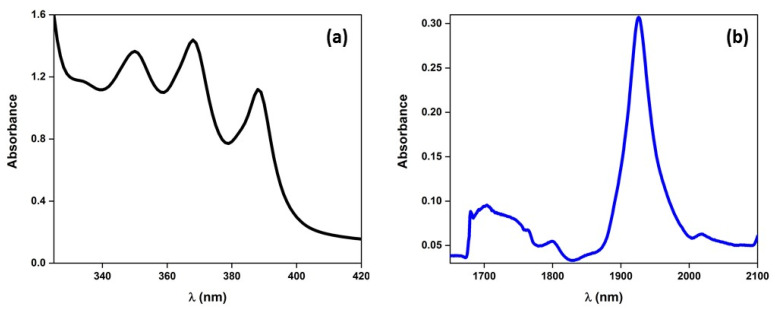
UV-Vis (**a**) and NIR (**b**) absorption spectra of the ZnMOF structure (1 mg/3 mL) in DMF.

To conduct an investigation of the process of absorption properties of the obtained ZnMOF structure, its behavior in the UV-Vis and NIR regions was investigated (Figure 5). The results clearly reflect a strong absorption from 347 to 386 nm. A meticulous structural investigation of the compound revealed that the significant and extensive UV-Vis absorption seen may be attributed to the absorption of H_2_L ligands and 1,2-bis(4-pyridyl) ethene. This can be attributed to the fact that the azo link serves as a proficient chromophore, capable of efficiently absorbing photoelectrons. The observed peak absorption at a wavelength of 366 nm can be attributed to the π–π* transition displayed by the π electrons of H_2_L.

Four different peaks were observed in the NIR absorption spectrum of ZnMOF. The strongest peak was observed at 1925 nm. The peak intensities at 1703 nm and 1800 nm are lower than the peak at 1925 nm. This observation provides evidence for the presence of a robust charge interaction inside the ZnMOF system, aligning with the findings obtained from the crystal structure study. The observed phenomenon can be attributed to the spatial restriction effect resulting from the inherent flexibility of ligands and the topology of the ZnMOF structure. The intense band at approximately 1925 nm in the NIR region in the framework is typical and is attributed to intervalence charge transfer (IVCT) within the Zn-H_2_L core. A lower-intensity band at 386 nm corresponds to weaker intramolecular charge transfer (ICT) in ZnMOF than the free ligand [71].

When ZnMOF was excited at a wavelength of 545 nm, it was observed that it had a maximum emission at 547 nm. This shows that the emission continues in the UV-Vis region, albeit at low intensity. In addition, while the absorption spectrum of H_2_L(MOF) (Figure S5A) is similar to that of ZnMOF, bpe(MOF) (Figure S5B) does not have significant absorption peaks in the UV-Vis region.

**Figure 6:**
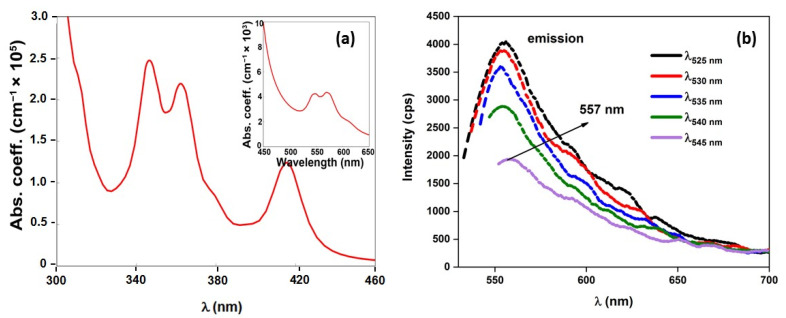
The absorption coefficient of ZnMOF as a function of wavelength obtained from a complex dielectric function (ε_2_(ω)) (**a**). Emission spectrum of ZnMOF excited at different wavelengths (**b**).

The calculated optical response has a high level of consistency with the experimental results (Figure 6a). The absorption onset is noticed at ~2 eV (~620 nm), in alignment with the electronic band gap, and the first absorption peak appears at 561 nm but is significantly smaller than the prominent peaks in the visible and NIR regions. In that sense, the absorption onset is not detected in the absorption spectrum measurements. Experimentally, when ZnMOF is excited with a wavelength of 545 nm, it has a spectrum with a maximum emission peak at 557 nm (Figure 6b). That is, although UV-Vis absorption is not clearly seen in the region, emissions continue to spread as a result of excitation at the maximum nm, which is in parallel with the calculated optical response. The theoretical and experimental absorption profiles are compatible with each other, and the small differences in peak positions originate from the fact that the calculations are based on an ideal ZnMOF structure without defects and deformations.

Fluorescence quantum yield and lifetime were calculated by excitation of anthracene, H_2_L, H_2_L(MOF), and ZnMOF at 360 nm (Table 2).

**Figure 7:**
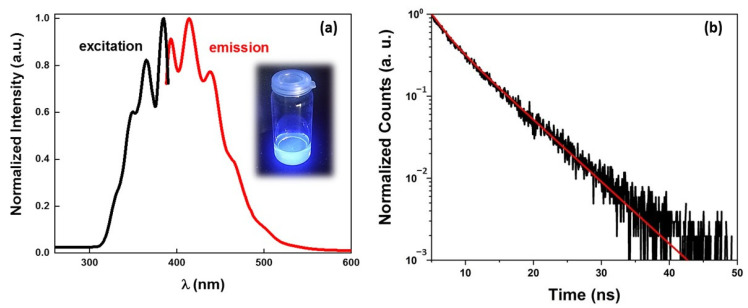
Fluorescence excitation (ε_abs_ 413 nm) and emission (ε_exc_ 366 nm) spectra of ZnMOF in ethanol at room temperature (**a**). The time-resolved emission decay curves of ZnMOF at a wavelength of 413 nm were measured under normal circumstances. The red line represents the fitting curves, while the black line represents the experimental data (**b**).

The shape of the excitation spectrum is nearly identical to the absorption spectrum of ZnMOF. Using the intersection of the fluorescence excitation (ε_abs_ 413 nm) and emission (ε_exc_ 366 nm) spectra of ZnMOF, the singlet excited state energy was calculated as 307.52 kJ mol^−1^ (Figure 7). Fluorescence quantum yield measurements were performed by using 9,10-diphenylanthracene (ϕ_f_ = 0.95 in ethanol) as a standard, and ϕ_f_ was found to be as high as 66.0%. According to the current understanding, the most significant blue emission from a Tb-MOF was documented in 2021. This emission occurred when the material was exposed to UV light at a wavelength of 365 nm, resulting in an exceptional quantum yield of 94.91% even in the presence of air [72].

It was observed that the fluorescence quantum yield is significantly increased with the bonding of the triazole ring on anthracene (Figure S6). While the binary MOF structure (bpe(MOF)) (Figure S7) consisting of ligand and Zn salt did not have fluorescence quantum efficiency and lifetime, the triple ZnMOF structure contributed somewhat to the increase in quantum efficiency.

**Figure 8:**
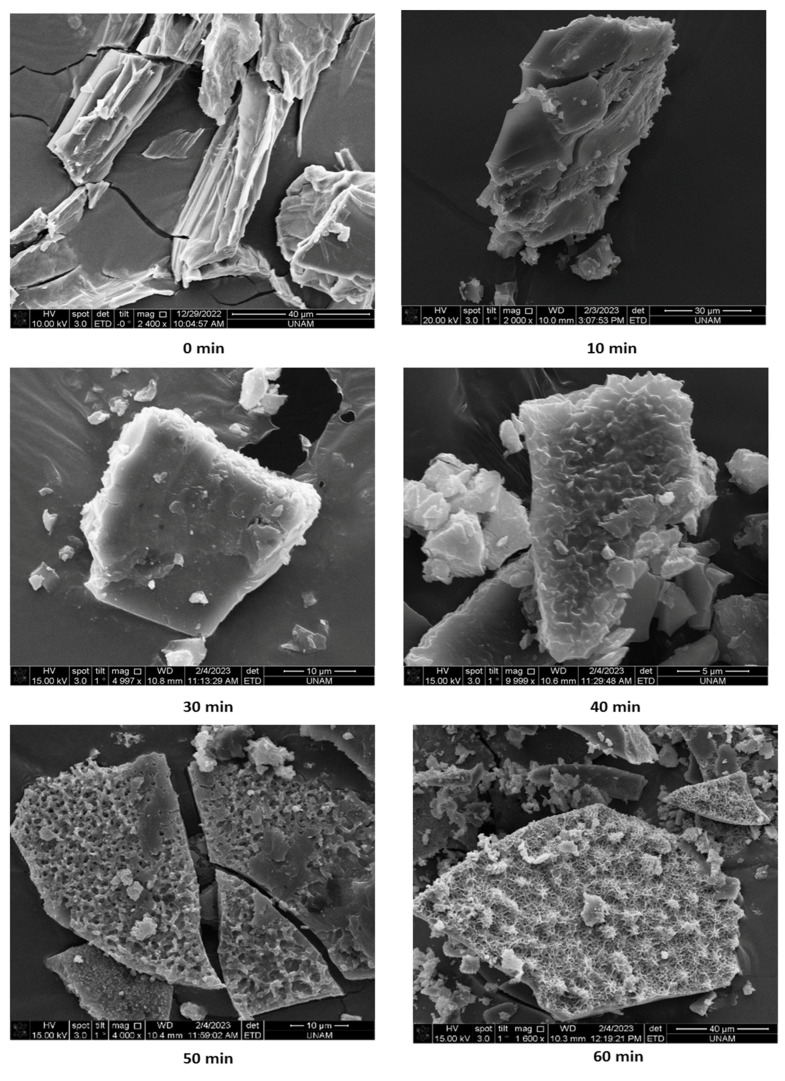
SEM images of ZnMOF formation from initial irradiation to 60 min of irradiation.

H_2_L and 1,2-bis(4-pyridyl)ethene with similar morphological structures started to react with zinc ion in the first 30 min. When the reaction reached 40 min, it was observed that the formation of floral structures began on the surface. The floral structures that are clearly seen at the 50 min mark completed their transformation at the 70 min mark (Figure 8).

**Figure 9:**
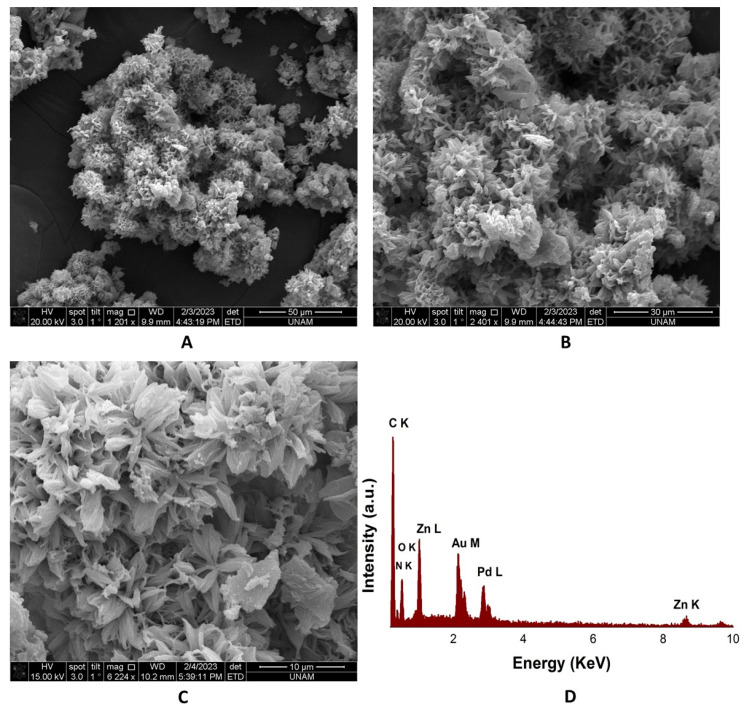
SEM images (**A**–**C**) and EDAX spectrum (**D**) of ZnMOF at the final stage (70 min).

In the synthesis made with the laser, microflowers continued throughout the 70 min reaction. Then, the growing microflowers overlapped at the end of 70 min. They formed macroscopically continuous films consisting of micro/nanoscopically acicular nanorods, as shown in Figure 9. The presence of pores on the surface of the active materials enhances the interfacial contact between the electrolyte and the material, reducing the diffusion path length for gas molecules and ions within the material [73,74].

The particle size determined using electron microscopy aligns with the findings of the X-ray diffraction pattern. Based on these research findings, compelling evidence suggests the occurrence of particle aggregation in samples consisting solely of ZnMOF. Consequently, the morphologies of these particles exhibit a high degree of uniformity. On the surface of ZnMOF, there are numerous open macropores with thin thickness, smooth wrinkles, and a diameter of approximately 1 µm. This unique 2D, layered, porous ZnMOF material exhibited ultra-low density, high specific surface area, and abundant functionality. The surface morphology of the ZnMOF was regular and contained the three-dimensional flowers consisting of approximately 2 µm sheets (Figure 9A,B). Smaller particle sizes can increase the accessibility of gases and ions and diminish the material’s diffusion distance. On the sheet structure of the obtained ZnMOF, as depicted in Figure 9C, a uniform flower structure forms. Also, the EDAX spectrum of ZnMOF shows weight % by element and atomic % by element (Figure 9D).

SEM images of binary MOF formations are quite different from the ZnMOF structure. While the formation of perforated layers is observed in the H_2_L(MOF) structure (Figure S8), SEM images of bpe(MOF) (Figure S9) consist of rectangular structures with smooth borders. These results show the difference in ZnMOF structure from binary MOF formations.

### 3.3. N_2_ Gas Adsorption

**Figure 10:**
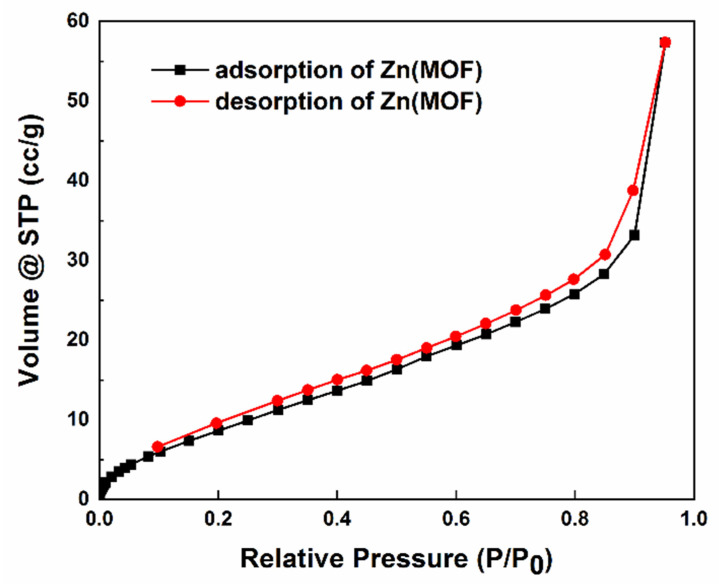
ZnMOF N_2_ adsorption and desorption isotherms.

The porosity and surface characteristics of nanoporous ZnMOF microspheres were explored by N_2_ adsorption/desorption analysis. The investigation of surface topology was conducted using Brunauer–Emmet–Teller (BET) surface area measurements. Figure 10 displays the BET adsorption isotherms of the analytes. In their study, Chen et al. present a synergistic sorbent separation technique that utilizes a packed-bed geometry to produce polymer-grade C_2_H_4_ in a single step from ternary (C_2_H_2_/C_2_H_6_/C_2_H_4_) or quaternary (CO_2_/C_2_H_2_/C_2_H_6_/C_2_H_4_) gas mixtures. Regenerating microporous metal-organic materials with extreme selectivity were synthesized, one of which exhibited a preference for C_2_H_6_ over CO_2_, C_2_H_2_, and C_2_H_4_ [75].

Figure 10 depicts the N_2_ adsorption and desorption isotherms of ZnMOF. These isotherms suggest that the interaction between adsorbent and adsorbate was weak, and the ZnMOF was loosely packed, forming wedge-shaped holes. The ZnMOF has a 40.345 m^2^g^−1^ surface area, an average pore volume of 0.013 cc/g, and average pore radius of 15.284 Å. Using the BJH method once more, the material’s pore radius distribution reveals three distinct pore types with radii of 15.3, 17.1, and 19.1 Å.

**Figure 11:**
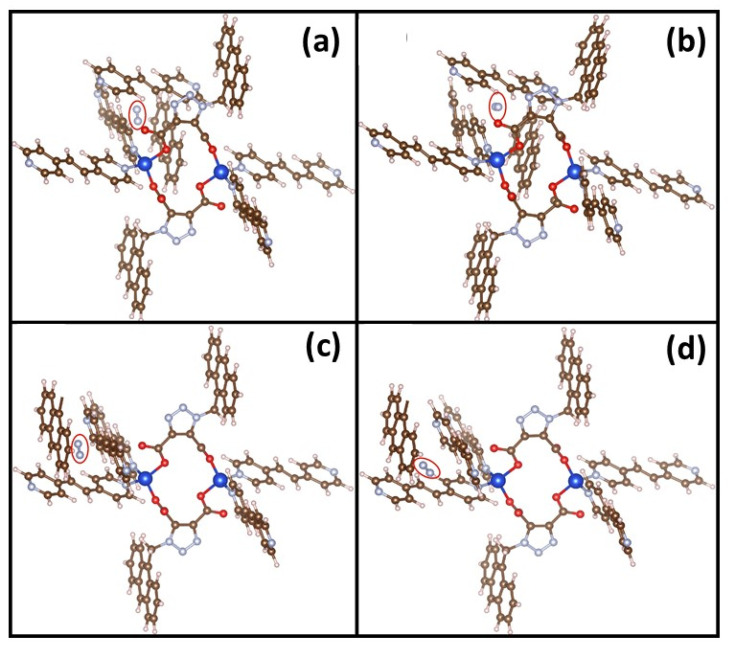
The possible adsorption (physisorption) sites for N_2_ (**a**–**c**) show initial and (**b**–**d**) final configurations.

In addition to BET analysis, the ab initio calculations confirm that N_2_ molecules can be efficiently physiosorbed by ZnMOF. There are multiple sites for N_2_ to settle, but the preferable locations are the interspaces between 1,2-bis(4-pyridyl)ethene ligands. Two possible adsorption sites are shown in Figure 11. The adsorption of N_2_ slightly distorts the structure (i.e., the distance between the ligands increases). However, the adsorption energy cannot be precisely calculated due to the structural deformation; the long bonding distance (>3 Å) and easy movement of the N_2_ molecules indicate physisorption (Videos S1–S3).

### 3.4. Pb Ion Uptake Experiments

In order to investigate the adsorption characteristics of ZnMOF, an aqueous solution containing Pb(II) ions was utilized for conducting adsorption tests. The objective of these tests was to analyze the impact of the initial concentration of Pb(II) ions on the absorption capacity of the adsorbent. The investigation focused on examining the impact of varying solution concentrations on the process of adsorption. A solution containing lead ions (Pb(II)) at a concentration of 1 mg mL^−1^ was generated by dissolving Pb(NO_3_)_2_. The preparation of all Pb(II) solutions involved the utilization of suitable sequential dilutions of the initial stock solution. Ten milliliters of lead solution was added to 3 mg of ZnMOF and mixed in a 50 mL tube at 160 rpm for 4 h. The pH value of the Pb(II) solution was recorded as 1.2. Then, the solution was separated from ZnMOF using centrifugation. Utilizing inductively coupled plasma-mass spectrometry (ICP-MS), the residual Pb(II) concentration in the solution was determined. The amount of adsorption at equilibrium, qe (mg g^−1^), and adsorption % (A%) were calculated according to Equations (1) and (2) [76,77].

(1) $$q_{e=\left(\frac{C_{0}-C}{m}\right)\times V}$$

(2) $$A\%=\left(\frac{C_{0}-C}{C_{0}}\right)\times 100$$

C_0_ (mg L^−1^) and C (mg L^−1^) are the initial and equilibrated concentrations of Pb(II) in Equations (1) and (2), respectively, V (L) is the volume of the solution, and m (g) is the mass of the dry ZnMOF.

The effect of concentration of Pb(II) adsorption on ZnMOF is shown in Table 3. The q_e_ and removal rate of the 50 ppm and 100 ppm lead ion solution by ZnMOF reached 6.04 mg/g and 38 mg/g, 3.02% and 9.5%, at 25 °C, respectively. It was found that the q_e_ and the removal efficiency were very low at a solution concentration of 50 mg/L, and the q_e_ and the adsorption efficiency of the Pb ion increased 6.29 times and 3.15 times by increasing the concentration to 100 mg/L at pH 1.2. In the experimental investigation, it was found that ZnMOF has better adsorption for 100 ppm lead solution when the concentration is increased.

This is because the presence of excess H^+^ at pH 1.2 competes with Pb(II) for adsorption sites on the adsorbent surface. As the Pb(II) concentration increases, the q_e_ value increases because the competition rate of the H^+^ ion decreases.

Zang and Li calculated the q_e_ values as 7.18 and 21.74 mg/g in their studies [78,79]. When the adsorption performance of ZnMOF was compared with references, ZnMOF demonstrated notable efficacy as an adsorbent, exhibiting potential for the efficient removal of Pb(II) from wastewater.

### 3.5. Photocatalytic Oxidative Degradation of MB with ZnMOF

**Figure 12:**
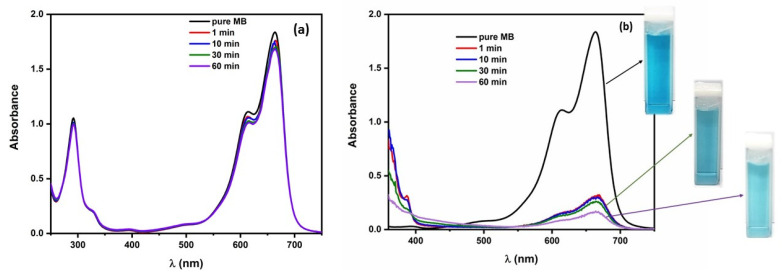
UV-Vis absorption spectrum of the photodegradation process of the pure MB solution (10 ppm) depending on the duration of irradiation (**a**). UV-Vis absorption spectrum of the photodegradation of the MB solution (10 ppm) with ZnMOF (2.5 mg/10 mL) depending on the irradiation time and the change of solution color (**b**).

A study was performed to determine the photocatalytic performance of ZnMOF, which was employed for the photodegradation of MB dye by exposing it to UV light and then using spectrophotometric monitoring to determine how much of the dye was broken down. The absorption spectra of pure MB without ZnMOF and MB in the presence of ZnMOF is recorded as a function of irradiation time, as shown in Figure 12. The observation reveals a rapid reduction in the intensity of the typical peak at 664 nm as the irradiation period progresses. The spectral bands detected within the visible range at wavelengths of 664 and 612 nm are indicative of the presence of chromophore groups inside the MB molecules, which can be attributed to the sulfur–nitrogen-conjugated system [80,81]. Consequently, the hue of the MB solution progressively diminished. Approximately 83% of the degradation of MB occurred within a 1 min period of irradiation in the solution containing ZnMOF. When the irradiation was extended to 60 min, a minimal alteration was detected.

The degradation percentage of MB dye, as presented in Table 4, was determined for irradiation durations of 1 min and 60 min using the following equation:

(3) $$Percentage\;of\;degradation=\frac{\left(C_{0}{-C}_{t}\right)}{C_{0}}\times 100$$

where C_0_ represents the initial concentration, and C_t_ represents the real-time concentration of MB.

**Figure 13:**
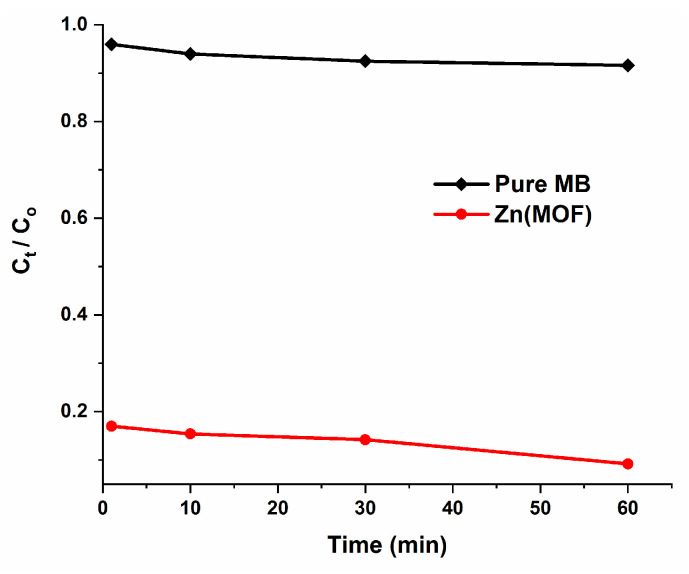
Photodegradation of the pure MB solution (10 ppm) (diamond) with ZnMOF (2.5 mg/10 mL) (circle).

Figure 13 illustrates the decolorization rate of MB through the utilization of polymeric nanocomposites. The graph presents the relative degradation (C_t_/C_0_) of MB as a function of time. Upon extending the duration of irradiation to 60 min, it was observed that the degradation percentage of MB reached 90.8. When comparing the outcomes achieved for the photocatalytic degradation of MB using ZnMOF, it becomes apparent that an irradiation time of 1 min is sufficient and yields even greater degradation levels.

As can be seen in Table 5, ZnMOF has a higher photodegradation ability than Zn-based MOFs in the literature.

**Figure 14:**
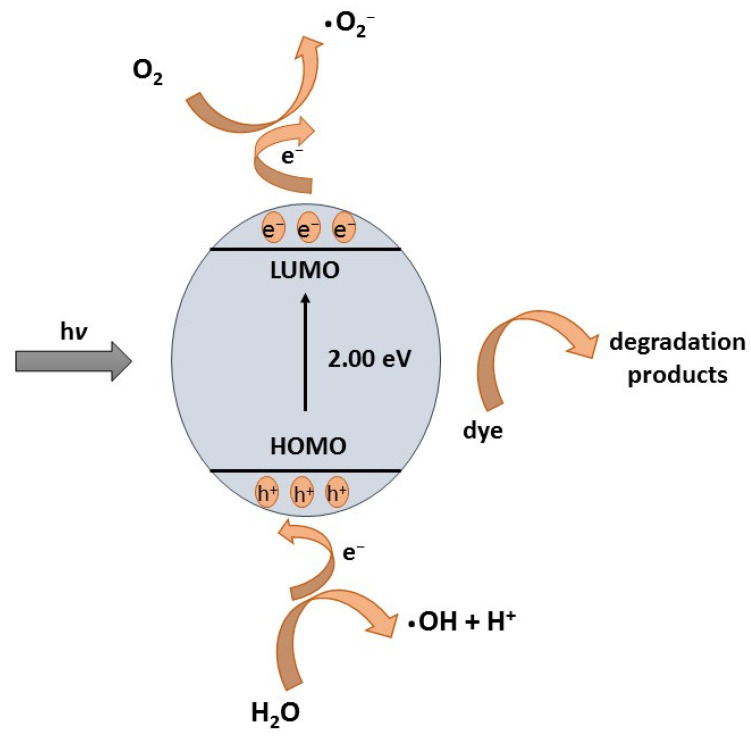
A simplified model of the mechanism of the photocatalytic reaction of MB in ZnMOF.

Typically, the electron of the excited state in the LUMO is readily lost, whereas the HOMO requires one electron to return to its stable state. Therefore, one electron is captured from the water molecules, and the ^.^OH active species is oxygenated. The ^.^OH radicals can then efficiently decompose MB to conclude the photocatalytic process. To comprehend the photocatalysis mechanisms of MOFs, the discrete nature of the light-induced transitions in MOFs should be described using the terminology of the HOMO–LUMO gap rather than the traditional semiconductor gap (CB-VB) (Figure 14) [84,85].

## 4. Conclusions

In this study, we reported a stable, reusable, and efficient photocatalytic Zn-based metal-organic framework (ZnMOF) with novel optical features that was synthesized via a new laser method. The suggested approach allows faster, more efficient, and more controllable MOF synthesis than solvothermal methods. The ZnMOFs were characterized using FTIR, SEM, and XRD analyses, which indicated the combination of amorphous and crystalline forms of the structure. The increase in the relative peak density of Zn-N and Zn-O in FTIR measurements is associated with increased ZnMOF content. Strong Zn-N stretching vibration and Zn-O stretching show evidence of the successful synthesis of ZnMOF. The absorption spectra in the UV-Vis and NIR regions of the synthesized ZnMOF structure were investigated, the active response at 387 nm in the UV-Vis region and at 1703, 1800, and 1925 nm in the NIR regions was revealed, and they were also confirmed with ab initio calculations. These features result in a photoluminescence behavior in the visible range with nanosecond relaxation time. Additionally, fluorescence quantum yield was measured to be high (66%), and lifetime was calculated to be long (1.8 and 5.7 ns). The Pb(II) adsorption performance of the system from lead ion solution was found to be approximately equal to other MOFs reported in the literature. Therefore, ZnMOF can be considered as a promising adsorbent for Pb(II) removal from wastewater. Finally, photocatalytic performance of ZnMOF was tested on photodegradation of MB dye, which was subjected to irradiation using UV light followed by spectrophotometric monitoring. The degradation percentage of MB was found to be 90.8%, and the obtained efficiency was larger than the reported values for other Zn-based MOFs. The proposed laser technology for MOF synthesis can inspire the development of a novel and competent platform for a fast MOF fabrication process and extend the possible applications of MOFs to miniaturized optoelectronic and photonic devices.

## Figures and Tables

**Table 1:** Comparison of the duration of the laser synthesis method and the solvothermal method of MOFs in the literature.

MOF Name	Synthesis Time (Minutes)	Synthesis Method	References
Zn(byia)	2880	Solvothermal	[65]
IRMOF-3	240	Autoclave reactor	[66]
ZnMOF	1440	Autoclave reactor	[67]
Zn_2_(BDC)_2_(DABCO)	300	Solvothermal	[68]
MOF-74	1440	Solvothermal	[69]
NiMOF	480	Oven	[70]
ZnMOF	70	Lazer induced	This study

**Table 2:** Performance comparison among various fluorescent materials.

	*ϕ*_em_^[a]^	τ/_ns_^[b]^
anthracene	33%	4.24
H_2_L	58%	1.9, 5.8
H_2_L(MOF)	63%	1.48, 6.0
ZnMOF	66%	1.8, 5.7

^[a]^ Fluorescence quantum yield. ^[b]^ Fluorescence lifetime (ns).

**Table 3:** Pb(II) adsorption values on ZnMOF.

Metal Ion Concentration, q_e_ (mg/g), and A% Values of Pb(II)			
C_0_ (mg/L)	C (mg/L)	q_e (mg/g)_	%Ads.
50	48.49	6.04	3.02
100	90.50	38	9.5

**Table 4:** The degradation percentage of MB dyes was determined after 60 min of irradiation.

Samples	Percentage of Degradation of MB Dye (1 min)	Percentage of Degradation of MB Dye (60 min)
Pure MB	2.6	9.2
ZnMOF	83	90.8

**Table 5:** Comparison of MB degradation studies.

Samples	Irradiation Time (min)	Efficiency %
ZnTCPc/UiO-66-Zr	120	68 [82]
Zn_2_(odpt)(bpy)(H_2_O)](bpy)_0.5_	60	74 [83]
ZIF-8	120	82.3 [16]
ZnMOF	60	90.8 present work

## Data Availability

The data presented in this study are available on request from the corresponding author.

## Supplementary Materials

The following supporting information can be downloaded at https://www.mdpi.com/article/10.3390/polym16020217/s1, Figure S1: FTIR spectrum of H_2_L(MOF) (a) and bpe(MOF) (b); Figure S2: XRD spectrum of zinc salt H_2_L and H_2_L(MOF) structures (a) and XRD spectrum of zinc salt, bpe, and bpe(MOF) (b), Figure S3: The 3D crystalline structure of Zn(MOF). The dark blue, light blue, red, brown, and white spheres represent Zn, N, O, C, and H atoms, respectively, Figure S4: The electronic band structure of ZnMOF along all symmetry directions, Figure S5: UV-Vis-NIR absorption spectrum in DMF (1 mg/3 mL) H_2_L(MOF) (a) and bpe(MOF) (b), Figure S6: Fluorescence excitation (ε_abs_ 413 nm) and emission (ε_exc_ 366 nm) spectrum of H_2_L (MOF) in ethanol at room temperature (a). Time-resolved emission decay curves of H_2_L (MOF) at 413 nm under ambient conditions, where the red line indicates the fitting curves, and the black line indicates the experimental data (b), Figure S7: Fluorescence excitation (ε_abs_ 355 nm) and emission (ε_exc_ 398 nm) spectrum of bpe(MOF) in ethanol at room temperature, Figure S8: SEM images of H_2_L (A,B) and H_2_L(MOF) at 70 min (C–F), Figure S9: SEM images of bpe (A,B) and bpe(MOF) at 70 min (C–F), Videos S1–S3: Movement of the N_2_ molecules.
