# Supplementary material for: Laser-Driven Rapid Synthesis of Metal-Organic Frameworks and Investigation of UV-NIR Optical Absorption, Luminescence, Photocatalytic Degradation, and Gas and Ion Adsorption Properties
Source: Polymers (Basel). 2024 Jan 12;16(2):217. doi: 10.3390/polym16020217 (PMC10820686; doi:10.3390/polym16020217)
Supplement: Supplementary file 1 [file polymers-16-00217-s001.zip › polymers-2743547-supplementary.pdf]

Supporting Information

# Laser-Driven Rapid Synthesis of Metal Organic Frameworks, Quantum Chemical Analysis and Investigation of UV-NIR Optical Absorption, Luminescence, Photocatalytic Degradation, and Gas and Ion Adsorption Properties

Saliha Mutlu 1,2, Bülend Ortaç 2,*, Dogukan Hazar Ozbey 2, Engin Durgun 2, Sevil Savaskan Yılmaz 1,2,* and Nergis Arsu 3,*

1 Department of Chemistry, Karadeniz Technical University, Trabzon 61080, Turkey; salihamutlu@ktu.edu.tr

2 National Nanotechnology Research Center (UNAM) and Institute of Materials Science Nanotechnology, Bilkent University, Ankara 06800, Turkey; hazar.ozbey@bilkent.edu.tr (D.H.O.); durgun@unam.bilkent.edu.tr (E.D.)

3 Department of Chemistry, Yildiz Technical University, Davutpasa Campus, Istanbul 34220, Turkey

* Correspondence: ortac@unam.bilkent.edu.tr (B.O.); sevily@ktu.edu.tr (S.S.Y.); narsu@yildiz.edu.tr (N.A.)

## Supplemental experimental procedures

## S.1. Experimental Section

### S.1.1. Synthesis of Lg-Based MOF (bpeMOF)

$Zn(NO_3).H_2O$ and 1,2-bis(4-pyridyl) ethane (bpe) (14.04 mg, 0.066 mmol) were mixed in solvents [$DMF/H_2O$ = 1:1 (v/v)]. The resulting solution was stirred for 5 minutes. Whitish in color bpeMOF crystals was synthesized in 70 minutes using a laser beam source at 88-90°C.

**S.1.2. Synthesis of $H_2L$ Based MOF ($H_2LMOF$)**

$Zn(NO_3)_2.6H_2O$ (10 mg, 0.033 mmol) and $H_2L$ (11.46 mg, 0.033 mmol) were mixed in solvents [DMF/$H_2O$ = 1:1 (v/v)]. The resulting solution was stirred for 5 minutes. Yellow colored $H_2LMOF$ crystals was synthesized in 70 minutes using a laser beam source at 88-90°C

**S.2. Material Characterization of Laser-Driven bpe(MOF) and $H_2L$(MOF)**

**2.1. Structure Characterization**

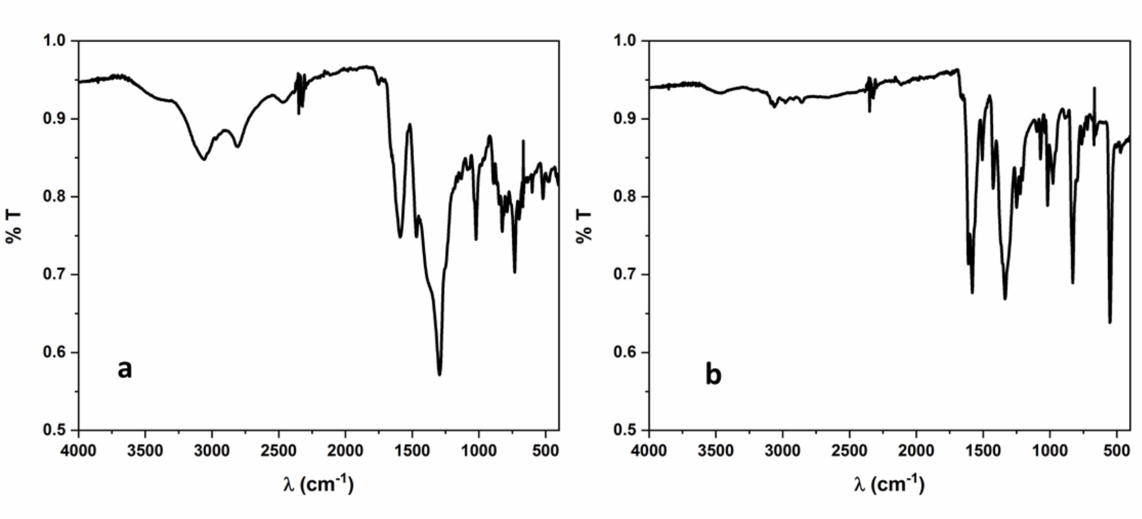


**Figure S1.** FTIR spectrum of $H_2L$(MOF) **(a)** and bpe(MOF) **(b)**

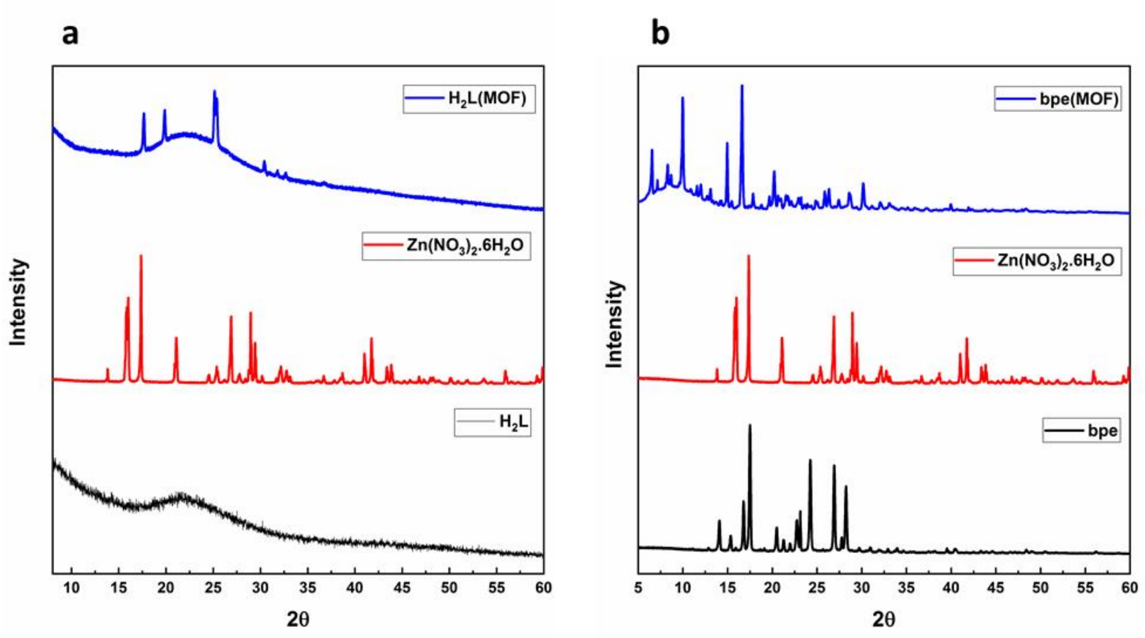


**Figure S2.** XRD spectrum of zinc salt, a $H_2L$ and $H_2L$(MOF) structures **(a)** XRD spectrum of zinc salt, bpe and bpe(MOF) **(b)**

It is seen that the crystal structure formation is more in bpe(MOF), while the amorphous structure of $H_2L$(MOF) is exhibited.

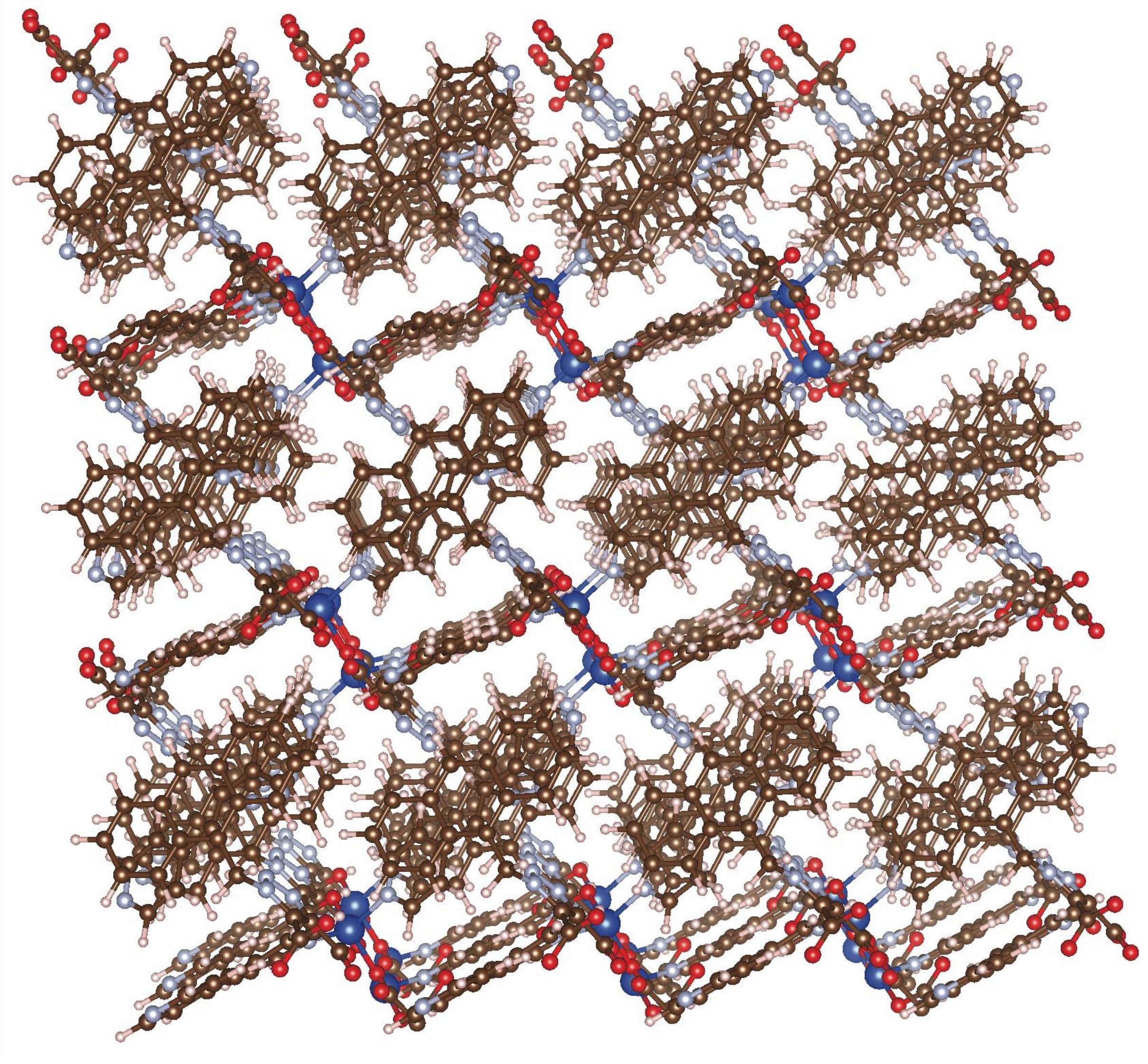

**Figure S3:** The 3D crystalline structure of ZnMOF. The dark blue, light blue, red, brown, and white spheres represent Zn, N, O, C, and H atoms respectively.

## 2.2. Photophysical Properties

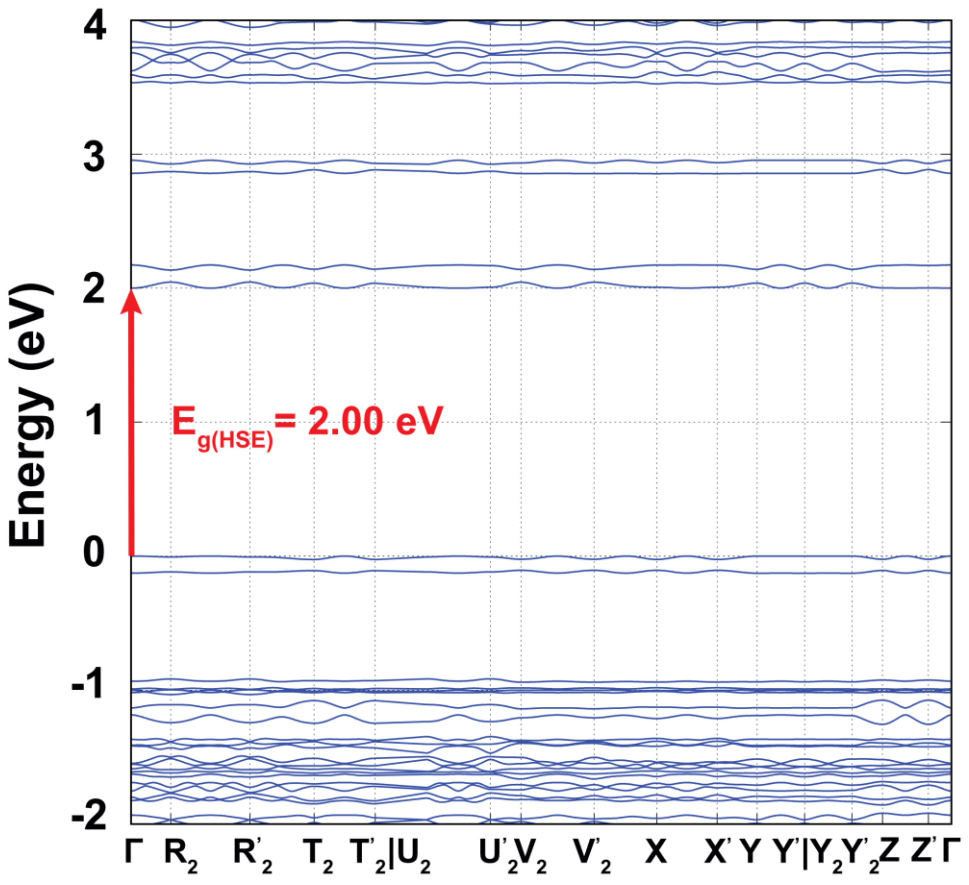


**Figure S4.** The electronic band structure of ZnMOF along all symmetry directions

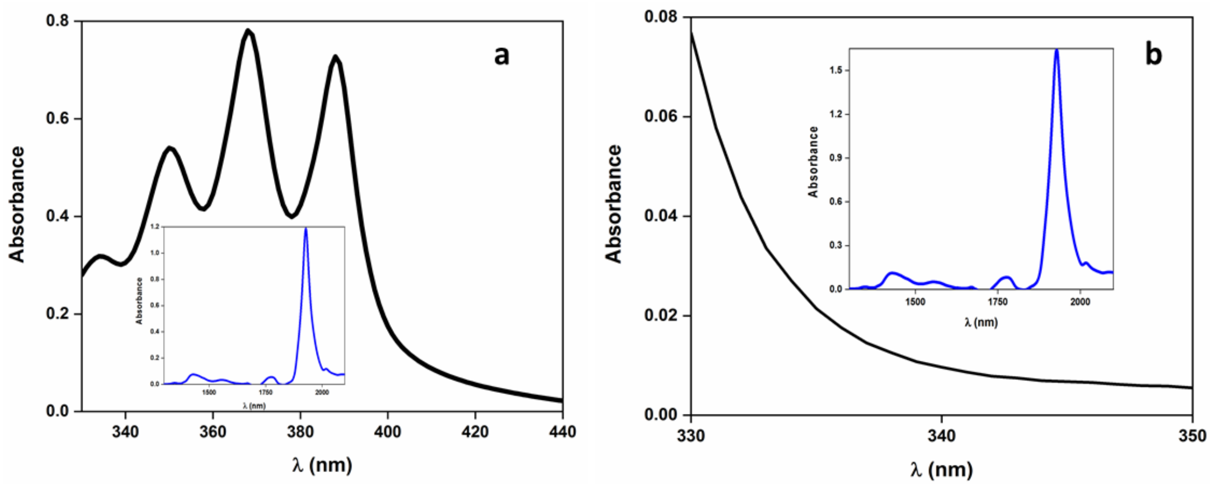


**Figure S5.** UV-Vis-NIR absorption spectrum in DMF (1mg/3ml) $H_2L$(MOF) **(a)** and bpe(MOF) **(b)**

In Figure S5B, while there are no absorption peaks in the Uv-vis region of BPE(MOF), the presence of absorption peaks belonging to both MOF structures in the NIR region is seen.

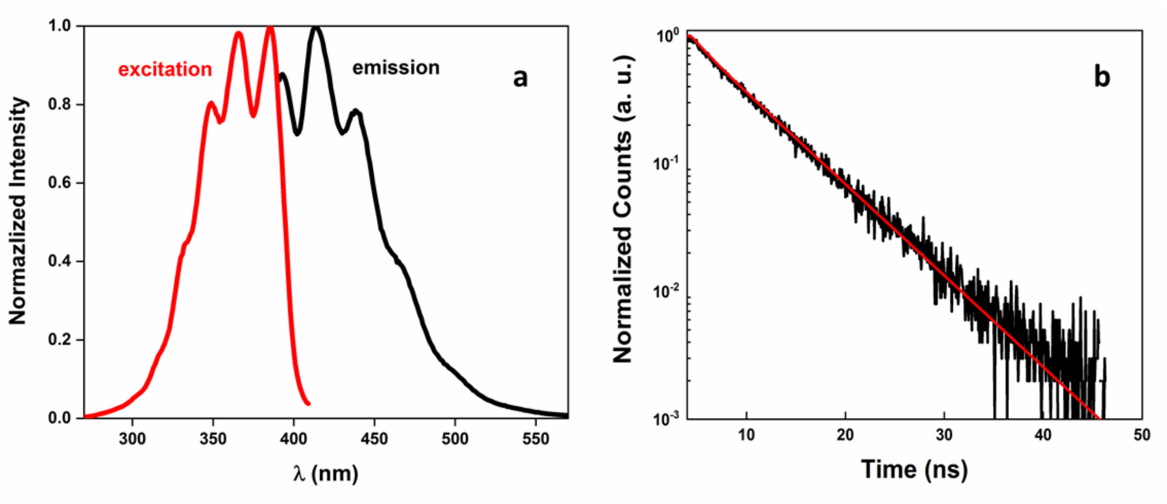


**Figure S6.** Fluorescence excitation ($\lambda_{obs}$ 413 nm) and emission ($\lambda_{exc}$ 366 nm) spectrum of $H_2L$ (MOF) in ethanol at room temperature **(a)**. Time-resolved emission decay curves of $H_2L$ (MOF) at 413 nm under ambient conditions, where the red line indicates the fitting curves and the black line indicates the experimental data **(b).**

The singlet excited state energy of $H_2L$(MOF) was estimated 306.7 kJ $mol^{-1}$. Fluorescence quantum yield measurement was performed by using of 9,10-diphenylanthracene ($\phi_f$ = 0.95 in ethanol) as standard and $\phi_f$ was found to be 63.0%. It also has fluorescence lifetimes of 1.4 and 6.0 ns.

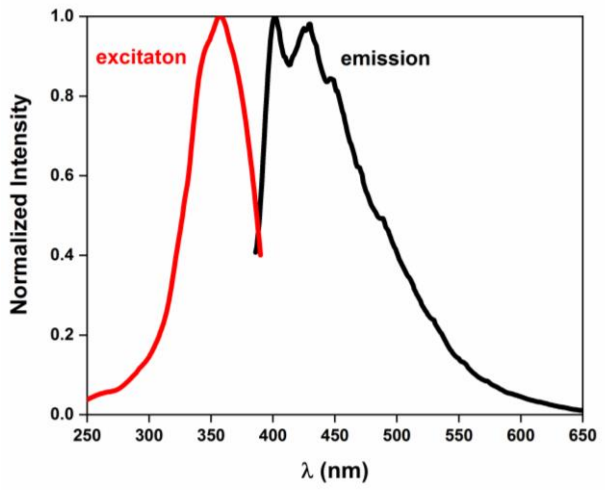


**Figure S7.** Fluorescence excitation ($\lambda_{abs}$ 355 nm) and emission ($\lambda_{exc}$ 398 nm) spectrum of bpe(MOF) in ethanol at room temperature

The singlet excited state energy of bpe(MOF) was estimated 309.11 kJ mol$^{-1}$. The ligand, which does not have photoluminescent chromophore groups, has no fluorescence quantum efficiency and lifetime of the metal organic framework structure.

## 2.3. Morphological Charactarization

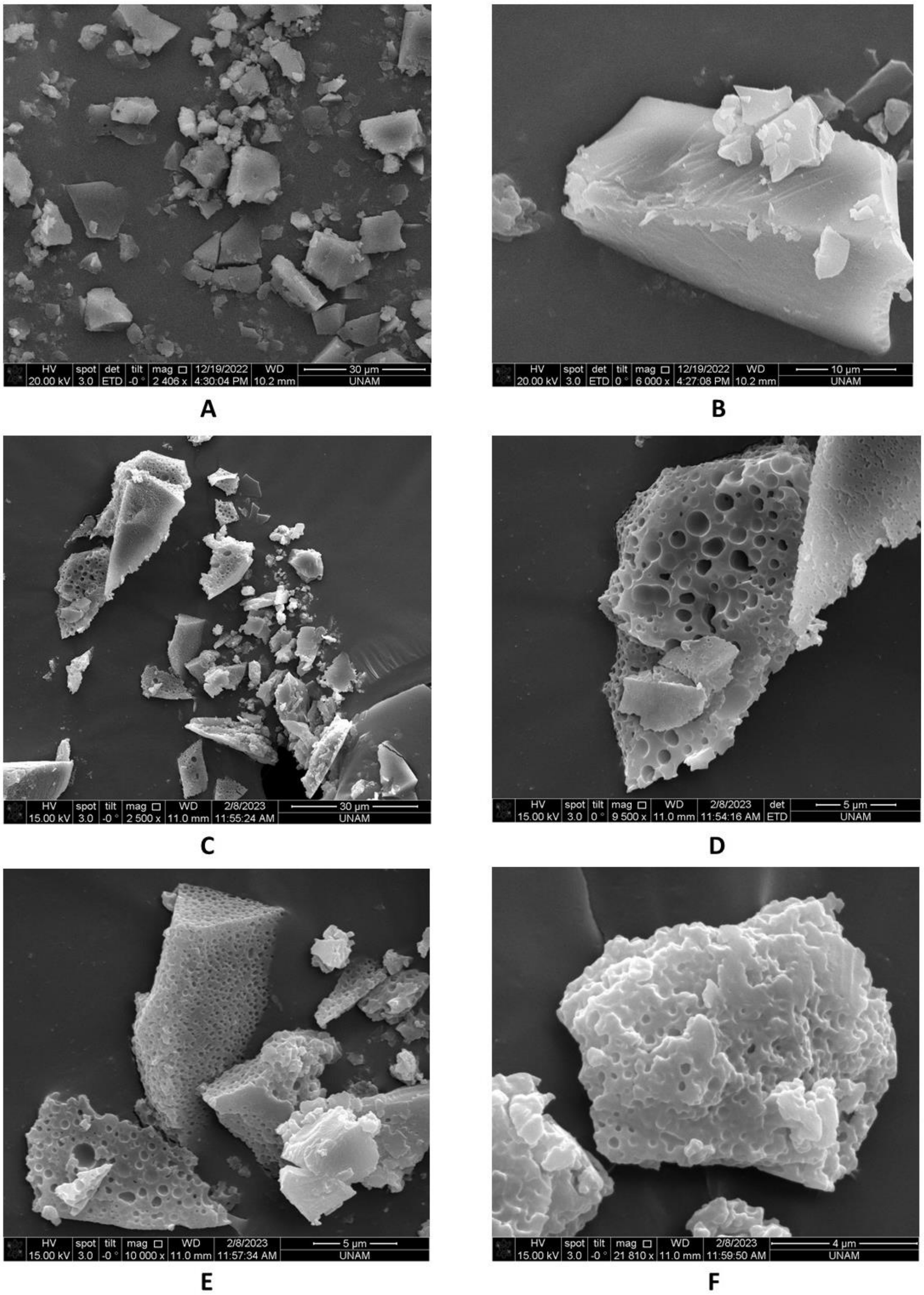


**Figure S8.** SEM images of $H_2L$ **(A,B)** and $H_2L$(MOF) at 70 min **(C-F)**

When the SEM images of $H_2L$(MOF) are examined, it is seen that the perforated structures with layers are formed.

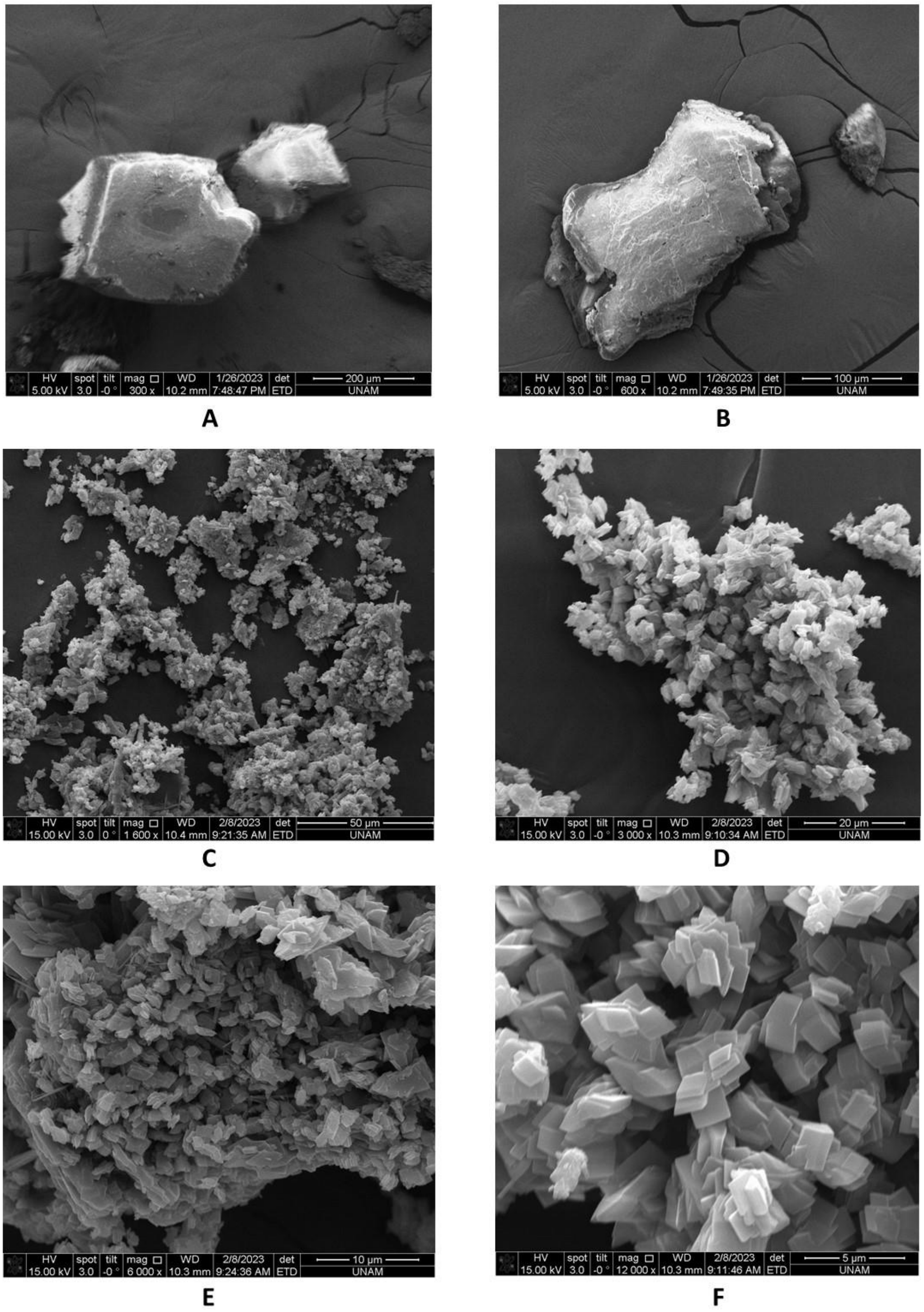


**Figure S9.** SEM images of bpe **(A,B)** and bpe(MOF) at 70 min **(C-F)**
